# Antiviral activities of peptide-based covalent inhibitors of the Enterovirus 71 3C protease

**DOI:** 10.1038/srep33663

**Published:** 2016-09-20

**Authors:** Yong Wah Tan, Melgious Jin Yan Ang, Qiu Ying Lau, Anders Poulsen, Fui Mee Ng, Siew Wen Then, Jianhe Peng, Jeffrey Hill, Wan Jin Hong, Cheng San Brian Chia, Justin Jang Hann Chu

**Affiliations:** 1 Institute of Molecular and Cell Biology, Agency for Science, Technology and Research (A*STAR), 61 Biopolis Drive, Proteos #06-05, 138673, Singapore; 2 Experimental Therapeutics Centre, Agency for Science, Technology and Research (A*STAR), 31 Biopolis Way, Nanos #03-01, 138669, Singapore; 3 Laboratory of Molecular RNA Virology and Antiviral Strategies, Department of Microbiology and Immunology, National University Health System, National University of Singapore 117597, Singapore

## Abstract

Hand, Foot and Mouth Disease is a highly contagious disease caused by a range of human enteroviruses. Outbreaks occur regularly, especially in the Asia-Pacific region, putting a burden on public healthcare systems. Currently, there is no antiviral for treating this infectious disease and the only vaccines are limited to circulation in China, presenting an unmet medical need that needs to be filled urgently. The human enterovirus 3 C protease has been deemed a plausible drug target due to its essential roles in viral replication. In this study, we designed and synthesized 10 analogues of the Rhinovirus 3 C protease inhibitor, Rupintrivir, and tested their 3 C protease inhibitory activities followed by a cellular assay using human enterovirus 71 (EV71)-infected human RD cells. Our results revealed that a peptide-based compound containing a trifluoromethyl moiety to be the most potent analogue, with an EC_50_ of 65 nM, suggesting its potential as a lead for antiviral drug discovery.

Hand, Foot and Mouth Disease (HFMD) is a self-limiting febrile illness, caused by a plethora of human enteroviruses, clinically characterized by vesiculo-papular rash on the hands, feet and mouth of afflicted individuals. The disease is highly contagious and outbreaks occur regularly in the Asia-Pacific region. In the years 2012 through 2014, China alone has seen annual outbreaks, with 2014 being the worst, with a total of 2.8 million reported cases and approximately 400 deaths. Normally manifesting as a mild illness in young children and immunocompromised adults, severe neurological complications like aseptic meningitis and poliomyelitis-like flaccid paralysis can develop in a minority of HFMD patients^1^,^2^. In particular, infections caused by human enterovirus 71 (EV71), have been associated with a higher incidence of severe HFMD manifestations^3^,^4^. Currently, there is neither an approved vaccine nor effective treatment regime for HFMD. Hence, it is of interest to develop new antiviral compounds against the common aetiological agents of the disease. Targeting viral enzymes essential for virus replication instead of host proteins is a strategy to develop therapeutics which is targeted to the viral pathogen involved with minimal cellular toxicity. The enterovirus genome encodes several enzymes, RNA-dependent RNA polymerase 3D, proteases 2 A and 3 C, which can serve as potential drug targets. The EV71 3 C is one of two proteases encoded by the viral genome, catalyzing the cleavage of the viral polyprotein at 8 different sites out of a total of 11^5^, making it an ideal target for drug intervention.

Rupintrivir (compound **1**; Table 1) is a Rhinovirus (RhV) 3C protease inhibitor which reached phase 2 clinical trials in 1999^6^. As rhinoviruses and enteroviruses are classified under the same genus, *Enterovirus*, their 3C proteases have high sequence and structural similarity. Recently, we conducted a structure-activity relationship (SAR) study using Rupintrivir and its analogues inhibiting the EV71 3C protease (strain 5865/sin/000009, GenBank accession no. **AF316321**) in a biochemical protease inhibition assay^7^. Rupintrivir inhibited the viral protease with an IC_50_ of 7.3 ± 0.8 μM^8^. In the same study, a Rupintrivir analogue, compound **2**, containing a structurally constrained phenylalanine moiety, was found to be 2-fold more potent than Rupintrivir (IC_50_ 3.2 ± 0.5 μM; Table 1). Herein, we describe further SAR studies of these analogues in conjunction with biochemical protease inhibition assay, antiviral cellular assay using human rhabdomyosarcoma (RD) cells and a parallel artificial membrane permeability assay (PAMPA), with the aim of developing cell-penetrating inhibitors using EV71-infected human cells.

## Results

### Flexibility at P3 residue of Rupintrivir analogue is important for antiviral activity in cells

Chemical structures, EV71 3C protease inhibitory activities, cellular antiviral activity assay using RD cells and cell permeability assay results (PAMPA) are tabulated in Table 1. Rupintrivir is a peptide substrate-based inhibitor exploiting the consensus recognition sequence of the Rhinovirus 3C protease. It can be divided into 4 components based on conventional nomenclature^9^, with the N-capping moiety (P4) furthest from the cleavage site, followed by P3, P2 and P1 residues respectively. The carboxyl terminal end of the P1 residue was modified to contain an ethyl propenoate moiety which targets the active cysteine residue in the 3C protease, forming an irreversible covalent bond to the cysteine’s sulfhydryl (–SH) group, inhibiting the target protease.

**Figure 1:**
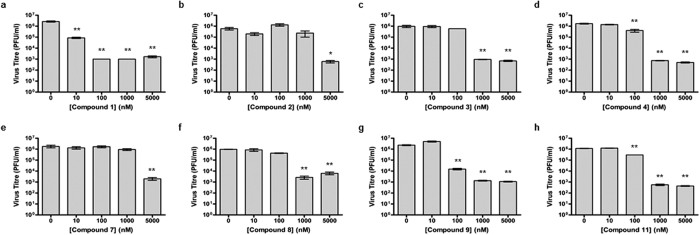
Synthesized analogues of Rupintrivir exhibited anti-viral activity to varying extents. Dose-dependent decrease in virus titre achieved by infected RD cells upon treatment of respective compounds at 0 nM (0.05% DMSO), 10 nM, 100 nM, 1 μM and 5 μM. Only compounds which exhibited anti-viral activity at or below 5 μM were shown. Statistical analysis was performed with one-way ANOVA (Dunnett’s method). **P ≤ 0.01, *P ≤ 0.05.

Rupintrivir (compound **1**; Table 1), has been shown to inhibit the EV71 3C protease with micromolar potency in earlier reports^5^,^10^. In our hands, Rupintrivir was found to possess an IC_50_ of 7.3 ± 0.8 μM in a biochemical protease inhibition assay^8^. Using the X-ray co-crystal structure of Rupintrivir and the EV71 3C protease^5^ followed by an SAR study, compound **2** (Table 1), a ring-constrained phenylalanine analogue was developed which was found to be 2-fold more potent than Rupintrivir in the biochemical protease inhibition assay (IC_50_ of 3.2 ± 0.5 μM)^8^. Intriguingly, when both compounds were introduced to EV71-infected human RD cells, only Rupintrivir was found to be capable of inducing a significant reduction in viral titre while compound **2** was weakly active (Fig. 1a,b). As the viral protease is localized to the viral replication complexes found on modified cellular membrane structures in the cytosol of infected cells^11^,^12^,^13^, the protease inhibitor must first traverse the cell membrane to exert its bioactivity. Suspecting that compound **2**’s poor activity was due to cell membrane penetration issues, both compounds were subjected to a PAMPA. Indeed, Rupintrivir was found to be highly membrane permeable while compound **2** was approximately 7-fold less permeable (P = 46.9 ± 4.7 × 10^−6^ *vs*. 7.1 ± 0.9 × 10^−6^ cm.s^−1^ respectively). We postulated that the highly rigid pyrrolidine ring at the P3 position was responsible for the membrane penetrating issues. Hence, the pyrrolidine ring scaffold was abandoned in our next series of inhibitors.

### Methylene moiety of Rupintrivir is required to retain membrane permeability and antiviral activity

Compound **3** (Table 1) was next synthesized with a 4-fluorophenylalanine in place of the constrained P2 phenylalanine. This compound exhibited similar potency to Rupintrivir (IC_50_s 6.2 ± 0.6 *vs*. 7.3 ± 0.8 μM respectively; Table 1). This, together with the absence of the rigid pyrrolidine scaffold, may have played a role in the observed improvement in its cellular antiviral activity compared to compound **2** (Fig. 1c) but inhibitor **3** was still significantly less potent than Rupintrivir in the antiviral cellular assay (Fig. 1a). We believe compound **3**’s ability to penetrate the cell membrane may have been compromised by the overall increased polarity due to the –NH– moiety between the P2 and P3 residues. For Rupintrivir, its –NH– moiety is replaced by a non-polar methylene (–CH_2_–), increasing its overall hydrophobicity and hence improving its propensity to penetrate cell membranes. Indeed, PAMPA results revealed that compound **3** was approximately 2-fold less permeable than Rupintrivir (P = 25.1 ± 3.6 × 10^−6^ *vs*. 46.9 ± 4.7 × 10^−6^ cm.s^−1^ respectively). In view of this, subsequent analogues reported in this paper were designed with a methylene moiety instead of a –NH– between the P2 and P3 residues.

### P4 moiety plays a role in cell permeability

In our previous paper, we showed that the EV71 viral 3C protease was highly specific for a P2 4-fluorophenylalanine and a P3 valine residue^8^, leaving limited avenues for further structural modifications at these two positions. An inspection of the X-ray co-crystal of Rupintrivir bound to the EV71 3C protease^5^ revealed that the protease S4 subsite pocket was considerably larger but shallow, bringing up the possibility that there may be further room for exploring P4 N-cap modifications. In addition, hydrogen bonding interactions between the 5-methylisoxazole-3-carbonyl P4 N-cap of Rupintrivir to Gly164 and Asn165 located in the S4 pocket suggested new N-capping groups can be designed to exploit potential hydrogen-bonding interactions within the S4 pocket. Using the X-ray co-crystal structure and molecular modeling, Rupintrivir’s P4 N-cap was replaced with a pyridazine moiety (compound **4**, Table 1). Our molecular model showed that the pyridazine C-2 aromatic nitrogen could participate in intramolecular hydrogen bonding to the P3 backbone NH while its C-3 aromatic nitrogen forms a hydrogen bond to the side-chain amide of Asn165, suggesting that the pyridazine moiety may be a plausible N-cap replacement. Indeed, protease inhibition assay results revealed compound **4**’s IC_50_ to be close to that of Rupintrivir (9.3 ± 0.6 *vs*. 7.3 ± 0.8 μM respectively; Table 1) but its antiviral activity on infected RD cells was significantly reduced (Fig. 1d). PAMPA results indicated that compound **4** suffered from a low propensity to penetrate the cell membrane compared to Rupintrivir (P = 12.6 ± 1.8 × 10^−6^ *vs*. 46.9 ± 4.7 × 10^−6^ cm.s^−1^ respectively) which may explain compound **4**’s poor antiviral activity. Replacing the N-cap with pyridines (compounds **5** and **6**; Table 1) either reduced or abrogated protease inhibition activity (IC_50_s 22.4 ± 3.5 and >100 μM respectively; Table 1). Expectedly, both compounds did not exhibit significant antiviral activity on infected RD cells (Table 1). Next, we modified the N-cap pyridazine moiety of compound **4** by methyl group attachment to increase hydrophobicity in an attempt to improve cell penetration (compounds **7** and **8**; Table 1). Protease inhibition assay results revealed compounds **7** and **8** (IC_50_s 5.2 ± 1.0 and 3.7 ± 0.6 μM respectively; Table 1) were marginally more potent than Rupintrivir and compound **4** (IC_50_s 7.3 ± 0.8 and 9.3 ± 0.6 μM respectively; Table 1) and this was attributed to hydrophobic interactions with the hydrophobic and aromatic side-chains of Ile125 and Phe170 in the 3C protease respectively. However, their antiviral activities on infected RD cells were still inferior compared to Rupintrivir (Fig. 1e,f). This suggested that the extra methyl group on the pyridazine ring did not significantly enhance cell penetration. PAMPA results supported this hypothesis, with compounds **7** and **8** (P = 11.6 ± 2.3 × 10^−6^ and 12.6 ± 0.7 × 10^−6^ cm.s^−1^ respectively) exhibiting similar membrane penetration properties to inhibitor **4** (P = 12.6 ± 1.8 × 10^−6^ cm.s^−1^), all significantly worse than Rupintrivir (P = 46.9 ± 4.7 × 10^−6^ cm.s^−1^). Taken together, the PAMPA results of compounds **4** to **8** suggested that the use of aromatic 6-membered rings for P4 N-capping may be detrimental to membrane penetration and this particular aromatic moiety was abandoned.

Further insights from the molecular modeling using non-aromatic alkyl P4 N-capping groups suggested that a trifluoromethyl (–CF_3_) moiety, a polar but hydrophobic group, could fit in the S4 subsite. Hence, compound **9** was synthesized containing a –CF_3_ P4 N-cap. In our biochemical protease inhibition assay, it was found to be equipotent to Rupintrivir (IC_50_s 7.7 ± 0.8 *vs*. 7.3 ± 0.8 μM respectively; Table 1) and exhibited antiviral activity on infected RD cells (Fig. 1g), significantly more potent than the inhibitors reported here so far. We attributed this to its membrane penetrating ability. Indeed, PAMPA results revealed that compound **9** possessed a much higher membrane penetrating propensity compared to compounds **2** to **8**, almost on par with Rupintrivir (P = 32.7 ± 6.7 × 10^−6^ *vs*. 46.9 ± 4.7 × 10^−6^ cm.s^−1^ respectively; Table 1). Interestingly, replacing the trifluoromethyl with a bulky *t*-butyl moiety (compound **10**) resulted in the abrogation of both biochemical and cellular activity (Table 1). We postulate this to be due to the *t*-butyl moiety being too large to fit the S4 pocket (IC_50_ >100 μM; Table 1). Replacing the *t*-butyl moiety with a less bulky *i*-propyl moiety (compound **11**) yielded a less potent inhibitor in the protease inhibition assay as compared to Rupintrivir (IC_50_ 11.7 ± 0.7 *vs*. 7.3 ± 0.8 μM respectively; Table 1), with significantly reduced antiviral activity (Fig. 1h). PAMPA results indicated that compound **11** was significantly less membrane permeable compared to Rupintrivir (P = 16.9 ± 0.8 × 10^−6^ *vs*. 46.9 ± 4.7 × 10^−6^ cm.s^−1^ respectively; Table 1) which may explain its lack of antiviral cellular activity.

### Compound 9 exhibits broad-spectrum antiviral activity against several Enteroviruses

**Figure 2:**
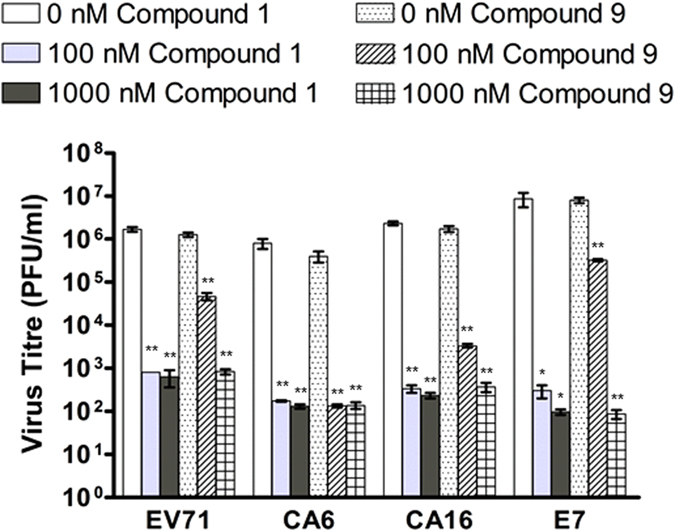
Both Compound 1 and Compound 9 demonstrated antiviral activities against Echovirus 7 (E7), Coxsackieviruses A6 (CA6) and A16 (CA16). Dose dependent decrease in virus titre was achieved by infected RD cells upon treatment of compounds 1 and 9 at 0 nM (0.05% DMSO), 100 nM and 1 μM. Statistical analysis was performed with one-way ANOVA (Dunnett’s method). **P ≤ 0.01.

In view of the antiviral activity of compound **9** demonstrated for EV71 infection, we sought to determine its efficacy against other HFMD-related enteroviruses to assess its potential as a possible lead for broad-spectrum anti-HFMD therapy. RD cells were infected with either Coxsackievirus A6 (CA6), Coxsackievirus A16 (CA16) (strain G-10, GenBank accession no. **U05876**), patient isolates of Coxsackievirus A6 (CA6), or Echovirus 7 (E7) and subjected to treatment with compound **9** at 100 nM or 1 μM or negative control 0.05% DMSO. Virus titres achieved after 12 hours of infection indicated that compound **9** exhibited potent antiviral activity against all tested viruses including EV71 (Fig. 2).

## Discussion

**Figure 3:**
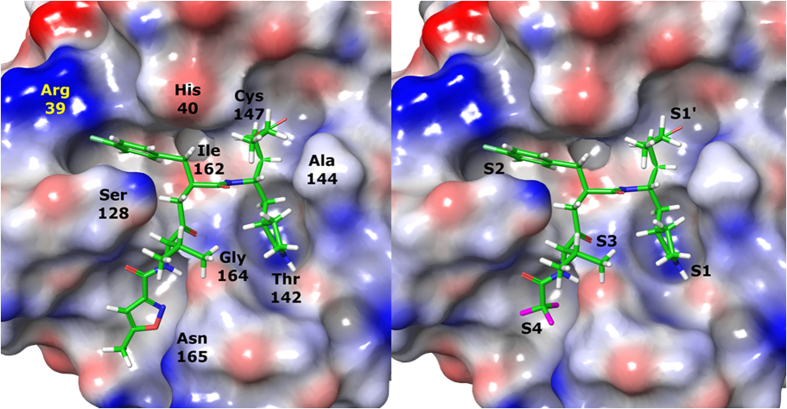
Structures of Rupintrivir and compound 9 at the EV71 3 C protease active site revealed minimal participation of S4 subsite in interactions with both compounds. Rupintrivir-EV71 3 C protease X-ray structure (left) and compound 9 modeled into EV71 (right). The protease is shown with an electrostatic surface and the inhibitor in tube with green carbon, red oxygen, blue nitrogen, magenta fluorine and white hydrogen. Key residues (left) and the binding subsites (right) are labeled.

In summary, 10 Rupintrivir analogues were designed and synthesized. We found that IC_50_ results from the biochemical assays were unreliable in predicting a compound’s antiviral activity. This is exemplified by the observation of compounds **2**, **7** and **8** showing greater potencies (IC_50_s) compared to Rupintrivir in the biochemical protease inhibition assay but failed to demonstrate significant antiviral activity when introduced to infected human RD cells (Table 1 and Fig. 1). We attribute this to the lack of cell-penetrating capability for the majority of the inhibitors reported (Table 1). Poor membrane permeability would have prevented the inhibitor from exerting its bioactivity on the EV71 3C protease. In addition, we discovered that the presence of an amide bond between the P2 and P3 residues was detrimental to cell penetration even though it demonstrated enhanced protease inhibition activity (compound **3**; Table 1). Our experiments also revealed that the nature of the P4 N-capping moiety could significantly influence a compound’s ability to penetrate the cell membrane as exemplified by the PAMPA and antiviral cellular assays. Lastly, we discovered that Rupintrivir’s isoxazole N-cap could be replaced with a trifluoromethyl moiety (compound **9**) with an EC_50_ (65 nM) close to the former (16 nM) for EV71 infections. Computer modeling was also used to compare the interactions between compounds **1** and **9** with EV71 3C protease, revealing the minimal participation of S4 pocket in the interaction between the compounds’ P4 residue and the protease active site, an affirmation of the P4 residue being critical for membrane permeability and not antiviral activity (Fig. 3). These findings together with the demonstration of efficacy against the other enteroviruses tested, opened new avenues for the design of new antiviral inhibitors for the treatment of EV71 infections.

## Methods

### Compound synthesis, purification and characterization

**Figure 4:**
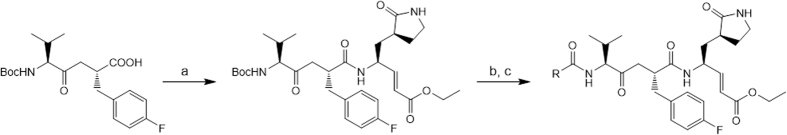
General synthetic procedure for compounds 4 to 11. Reagents and conditions: **(a)** (*S*,*E*)-ethyl 4-amino-5-(*S*-2-oxopyrrolidin-3-yl)pent-2-enoate, DIC, HOBT, DMF, 25 °C, 16 h.; **(b)** 95% TFA in CH_2_Cl_2_ (v/v), 25 °C, 30 min.; **(c)** appropriate carboxylic acid, PyClock, DIPEA, DMF, 25 °C, 4 h.

All reagents and solvents were obtained from commercial sources and were used without further purification. Rupintrivir (Compound **1**) was purchased from Santa Cruz Biotechnology Inc. (USA). Compounds **2** and **3** were synthesized based on a previously reported protocol (Ang et al., 2015). (2*R*,5*S*)-5-(*t*-butoxycarbonyl amino)-2-(4-fluorobenzyl)-6-methyl-4-oxoheptanoic acid and (*S*,*E*)-ethyl 4-amino-5-(*S*-2-oxopyrrolidin-3-yl)pent-2-enoate was custom-made by Wuxi Apptec (China). Crude compounds were purified using a reverse-phase C18 column on a high performance liquid chromatography (HPLC) system with an ultraviolet detector set at 215 nm. The mobile phase consisted of solvent A (water) and solvent B (acetonitrile). The gradient started with 45% solvent B for 5 min which was increased to 75% in 40 min. All target compounds were characterized by electrospray ionization mass spectrometry. Samples were dissolved in 50% acetonitrile containing 0.1% formic acid and directly injected via a syringe pump at 5 μLmin-1. The mass spectra were acquired at 120,000 FWHM with internal calibration. NMR spectra were recorded on a 400 MHz spectrometer in CD_3_OD. Chemical shifts were expressed as δ (ppm) relative to tetramethylsilane (TMS). The general synthetic scheme for Compounds **4** to **11** is summarized in Fig. 4(a) (S,E)-ethyl 4-amino-5-(*S*)-2-oxopyrrolidin-3-yl)pent-2-enoate, DIC, HOBT, DMF, 25 °C, 16 h.; (b) 95% TFA in CH_2_Cl_2_ (v/v), 25 °C, 30 min.; (c) appropriate carboxylic acid, PyClock, DIPEA, DMF, 25 °C, 4 h.

The general synthetic protocol is as follows: (a) (2*R*,5*S*)-5-(*t*-butoxycarbonylamino)-2-(4-fluorobenzyl)-6-methyl-4-oxoheptanoic acid (1.37 g, 3.6 mmol, 1.2 eq.) and (*S*,*E*)-ethyl-4-amino-5-(*S*)-2-oxopyrrolidin-3-yl)pent-2-enoate (0.68 g, 3.0 mmol, 1 eq.) were dissolved in DMF (20 mL) and stirred at 25 °C, 20 min. DIC (1.14 g, 9.0 mmol, 3 eq.) and HOBT (0.08 g, 0.6 mmol, 0.2 eq.) were then added and the reaction left to react at 25 °C, 16 h. Upon completion, the reaction was quenched by the addition of water and the aqueous layer was extracted thrice with ethyl acetate (3 × 10 mL) and the combined organic layers were washed with saturated brine and concentrated under reduced pressure to give the Boc-protected intermediate as a yellow oil. (b) 95% TFA in CH_2_Cl_2_ v/v (6 mL) was added to the yellow oil and left to react at 25 °C, 30 min. Excess TFA was removed by a stream of N_2_ (g) to obtain a brown oil which was subsequently purified by column chromatography (CH_2_Cl_2_ and CH_3_OH solvents) and dried *in vacuo* to obtain the unprotected intermediate as an off-white powder (0.73 g, 1.5 mmol, 50% overall yield). (c) The intermediate (0.1 g, 0.2 mmol, 1 eq.), DIPEA (0.13 g, 1.0 mmol, 5 eq.) and the appropriate N-capping carboxylic acid (1.0 mmol, 5 eq.) were dissolved in DMF (5 mL). PyClock (2.77 g, 5.0 mmol, 5 eq.) was added and the reaction mixture was stirred at 25 °C, 4 h. The reaction was quenched by the addition of water and the crude product was extracted thrice with ethyl acetate (3 × 10 mL). The combined organic layers were washed with saturated brine and concentrated under reduced pressure. The crude material was purified by HPLC (H_2_O and CH_3_CN solvent) and dried *in vacuo* to obtain the target products as colorless gels with overall yields of 3 to 8%. Compound characterization information:

Compound **4**, ethyl-(*S*,*E*)-4-(2*R*,5*S*)-2-(4-fluorobenzyl)-6-methyl-4-oxo-5-pyridazine-3-carboxamidoheptanamido-5-(*S*)-2-oxopyrrolidin-3-yl-pent-2-enoate: ^1^H NMR (400 MHz, CD_3_OD) δ 0.96 (6H, d, *J* = 6.9 Hz), 1.29 (3H, t, *J* = 7.3 Hz, ester), 1.46-3.34 (13H, m), 4.16-4.70 (4H, m), 5.37, 6.61 (2H, d, *J* = 15.4 Hz, *E*-alkene), 6.62-9.34 (7H, m, aromatics). ESI-MS: *m/z* calc C_31_H_39_FN_5_O_6_ (M + H^+^) 596.2884, found 596.2883.

Compound **5**, ethyl-(*S*,*E*)-4-(2*R*,5*S*)-2-(4-fluorobenzyl)-6-methyl-4-oxo-5-picolinamidoheptanamido-5-(*S*)-2-oxopyrrolidin-3-yl-pent-2-enoate: ^1^H NMR (400 MHz, CD_3_OD) δ 0.96 (6H, d, *J* = 6.9 Hz), 1.29 (3H, t, *J* = 7.3 Hz, ester), 1.39-3.38 (13H, m), 4.10-4.68 (4H, m), 5.35, 6.61 (2H, d, *J* = 15.7 Hz, *E*-alkene), 6.87-8.71 (8H, m, aromatics). ESI-MS: *m/z* calc C_32_H_40_FN_4_O_6_ (M + H^+^) 595.2931, found 595.2928.

Compound **6**, ethyl-(*S*,*E*)-4-(2*R*,5*S*)-2-(4-fluorobenzyl)-6-methyl-5-nicotinamido-4-oxoheptanamido-5-(*S*)-2-oxopyrrolidin-3-yl-pent-2-enoate: ^1^H NMR (400 MHz, CD_3_OD) δ 0.97 (6H, d, *J* = 6.9 Hz), 1.28 (3H, t, *J* = 7.3 Hz, ester), 1.39-3.67 (13H, m), 4.05-4.70 (4H, m), 5.36, 6.62 (2H, d, *J* = 15.6 Hz, *E*-alkene), 6.82-9.05 (8H, m, aromatics). ESI-MS: *m/z* calc C_32_H_40_FN_4_O_6_ (M + H^+^) 595.2931, found 595.2928.

Compound **7**, ethyl-(*S*,*E*)-4-(2*R*,5*S*)-2-(4-fluorobenzyl)-6-methyl-5-(4-methylpyridazine-3-carboxamido)-4-oxoheptanamido-5-(*S*)-2-oxopyrrolidin-3-yl-pent-2-enoate: ^1^H NMR (400 MHz, CD_3_OD) δ 0.98 (6H, d, *J* = 6.9 Hz), 1.29 (3H, t, *J* = 7.1 Hz, ester), 1.38-3.40 (16H, m), 4.05-4.67 (4H, m), 5.37, 6.62 (2H, d, *J* = 16.0 Hz, *E*-alkene), 6.86-9.19 (6H, m, aromatics). ESI-MS: *m/z* calc C_32_H_41_FN_5_O_6_ (M + H^+^) 610.3040, found 610.3038.

Compound **8**, ethyl-(*S*,*E*)-4-(2*R*,5*S*)-2-(4-fluorobenzyl)-6-methyl-5-(6-methylpyridazine-3-carboxamido)-4-oxoheptanamido-5-(*S*)-2-oxopyrrolidin-3-yl-pent-2-enoate: ^1^H NMR (400 MHz, CD_3_OD) δ 0.98 (6H, d, *J* = 6.9 Hz), 1.29 (3H, t, *J* = 7.1 Hz, ester), 1.38–3.39 (16H, m), 4.08–4.71 (4H, m), 5.37, 6.61 (2H, d, *J* = 16.3 Hz, *E*-alkene), 6.86–8.23 (6H, m, aromatics). ESI-MS: *m/z* calc C_32_H_41_FN_5_O_6_ (M + H^+^) 610.3040, found 610.3041.

Compound **9**, ethyl-(*S*,*E*)-4-(2*R*,5*S*)-2-(4-fluorobenzyl)-6-methyl-4-oxo-5-(2,2,2-trifluoro acetamidoheptanamido-5-(*S*)-2-oxopyrrolidin-3-yl-pent-2-enoate: ^1^H NMR (400 MHz, CD_3_OD) δ 0.97 (6H, d, *J* = 6.8 Hz), 1.23 (3H, t, *J* = 7.1 Hz, ester), 1.32-3.21 (13H, m), 4.04-4.54 (4H, m), 5.43, 6.70 (2H, d, *J* = 15.8 Hz, *E*-alkene), 7.05, 7.20 (4H, m, aromatics). ESI-MS: *m/z* calc C_28_H_36_F_4_N_3_O_6_ (M + H^+^) 586.2540, found 586.2540.

Compound **10**, ethyl-(*S*,*E*)-4-(2*R*,5*S*)-2-(4-fluorobenzyl)-6-methyl-4-oxo-5-pivalamidoheptanamido-5-(*S*)-2-oxopyrrolidin-3-yl-pent-2-enoate: ^1^H NMR (400 MHz, CD_3_OD) δ 0.97 (6H, d, *J* = 6.8 Hz), 1.23 (3H, t, *J* = 7.1 Hz, ester), 1.88 (9H, s), 2.11-3.35 (13H, m), 4.00-4.91 (4H, m), 5.45, 6.68 (2H, d, *J* = 15.5 Hz, *E*-alkene), 7.07, 7.29 (4H, m, aromatics). ESI-MS: *m/z* calc C_31_H_45_FN_3_O_6_ (M + H^+^) 574.3292, found 574.3290.

Compound **11**, ethyl-(*S*,*E*)-4-(2*R*,5*S*)-2-(4-fluorobenzyl)-5-isobutyramido-6-methyl-4-oxoheptanamido-5-(*S*)-2-oxopyrrolidin-3-yl-pent-2-enoate: ^1^H NMR (400 MHz, CD_3_OD) δ 0.72-1.02 (12H, m), 1.22 (3H, t, *J* = 7.1 Hz, ester), 1.33-3.19 (14H, m), 3.92-4.18 (4H, m), 5.45, 6.69 (2H, d, *J* = 15.6 Hz, *E*-alkene), 7.05, 7.18 (4H, m, aromatics). ESI-MS: *m/z* calc C_30_H_43_FN_3_O_6_ (M + H^+^) 560.3136, found 560.3134.

### Protease inhibition assay

EV71 3C protease inhibition assays were based on a published procedure^7^ and performed in a buffer containing Tris-HCl (50 mM), NaCl (150 mM), EDTA (1 mM), glycerol (10% v/v) and DTT (2 mM) at pH 7.0. The protease (6 μM) and varying inhibitor concentrations were incubated at 25 °C for 2 h. The final DMSO concentration was maintained at 2%. After that, the chromogenic peptide substrate succinyl-EALFQ-pNA (Peptides International, USA) was added to make a final concentration of 200 μM. The contents were incubated at 25 °C for 2 h. Absorbance at 405 nm was measured with a plate reader at 30 °C. All experiments were conducted in duplicates. IC_50_ values were derived by fitting the initial velocity against the log [inhibitor] using GraphPad Prism 5 software (USA).

### Virus infection and plaque assay

Human RD cells were cultured in DMEM/F-12 (Gibco) supplemented with 10% FBS (GE Healthcare) and the infection medium used for all infections and compound treatment contained was supplemented with 2% FBS. For the compound treatment assays, monolayers of RD cells were first infected with EV71 at a multiplicity of infection (MOI) of 1 for 1 hour at 37 °C, 5% CO_2_. The compounds were then introduced to the cells at different concentrations. Culture supernatant was collected at 12 hours post-treatment for determination of infectious virus titre by viral plaque assay. Each culture supernatant was 10-fold serially diluted and 100 μL was added, in triplicates, to a monolayer of RD cells in a 24-well format. The infection was allowed to proceed for 1 h at 37 °C, 5% CO_2_ before the virus was removed. The cells were then washed to remove unbound virus particles with PBS (pH 7.4) and overlaid with infection medium containing 0.5% agarose. The cells were incubated for 48 hours at 37 °C before they were fixed with 4% paraformaldehyde and stained with crystal violet. Plaques that formed were counted visually and the infectious virus titre was calculated, expressed as the average number of PFU per milliliter (PFU/mL) of sample. The 50% effective concentration, EC_50_, was determined for selected compounds by a non-linear regression graph fitting of the virus titre obtained at a minimum of 5 different compound concentrations and 0.05% DMSO was included as an untreated control.

### Cell viability assay

Cell viability assay was conducted for all compounds using AlamarBlue^®^ reagent (Invitrogen). RD cells subjected to compound treatment under the same conditions as the experimental samples for 12 hours was incubated with 10% AlamarBlue^®^ reagent in infection medium for 2 h at 37 °C, 5% CO_2_. Cells treated with 0.05% DMSO were used as the reference and percentage cell viability was determined for each compound concentration (Supplementary Figure S1). Compounds which maintained a cellular viability of ≥80% were deemed to be non-cytotoxic to RD cells.

### Parallel artificial membrane permeability assay (PAMPA)

PAMPA was conducted by GVK Biosciences (India) using Pion PAMPA Sandwich Plates (Pion Inc., USA). Briefly: test compounds were dissolved in buffer at pH 7.4 to make a 50 μM stock solution. 200 μL was then added to the donor compartment of the plate. The plate was inverted and 200 μL of buffer (pH 7.4) was added to the acceptor compartment. After incubation (4 hours), a 50 μL sample was analyzed by UV using a spectrophotometer. Experiments were conducted in triplicates. Compound permeability (P) was calculated using the formula:


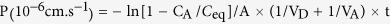


where: C_A_ = compound concentration in the acceptor compartment at 4 h, C_eq_ = compound concentration at equilibrium at 4 h, A = filter area (0.3 cm^2^), V_A_ = acceptor compartment volume (200 μL), V_D_ = donor compartment volume (200 μL), t = incubation time (14,400 s). Compounds with a P value of <10 × 10^−6^ cm.s^−1^ are considered to have low permeability.

### Molecular modeling

The EV71 protease X-ray structure (PDB accession no. **3SJO**) was prepared with the Protein Preparation Wizard in Maestro 9.3 (Schrödinger, USA) using standard settings. This included the addition of hydrogen atoms, bond assignments, removal of water molecules >7 Å from the ligand, protonation state assignment, optimization of the hydrogen bond network and restrained minimization using the OPLS2005 force field^14^. The co-crystallized, covalently-bound inhibitor was used as a template for modelling the conformation and orientation for all inhibitors listed in this paper. The inhibitor-protein complex was finally energy-minimized using Macromodel 9.9 (Schrödinger, USA). All residues >9 Å from the ligand were constrained before the complex was subjected to 500 steps of Conjugate Gradient energy minimization using the OPLS2005 force field^15^ and GB/SA continuum solvation method^16^. Model visualization was done using Maestro v 9.3.

## Additional Information

**How to cite this article**: Tan, Y. W. *et al.* Antiviral activities of peptide-based covalent inhibitors of the Enterovirus 71 3C protease. *Sci. Rep.* **6**, 33663; doi: 10.1038/srep33663 (2016).

## Supplementary Material

Supplementary Information

## Figures and Tables

**Table 1:** Summary Table of structure of inhibitors, EV71 3 C protease inhibition potencies and cell-based antiviral activities.

Cpd. #	Structure	Inhibitory Potency, IC_50_ (µM)	Cell-based EV71 Inhibitory Activity	Permeability (P) at pH 7.4 (10^−6^ cm.s^−1^)
1	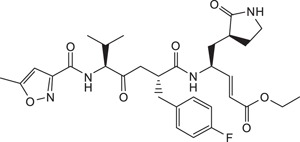	7.3±0.8	++++	46.9±4.7
2	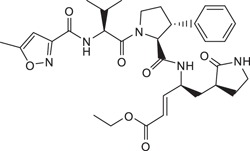	3.2±0.5	+	7.1±0.9
3	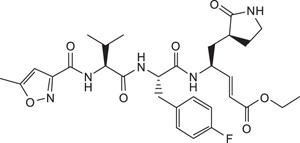	6.2±0.6	++	25.1±3.6
4	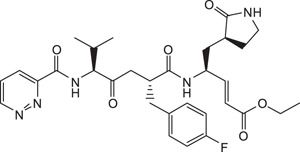	9.3±0.6	++	12.6±1.8
5	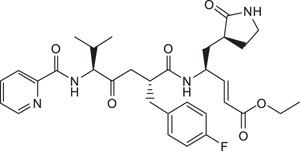	22.4±3.5	0	14.9±3.3
6	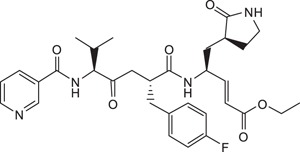	> 100	0	16.9±5.2
7	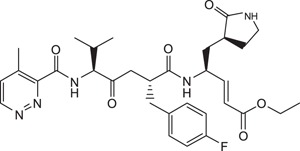	5.2±1.0	+	11.6±2.3
8	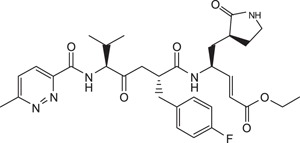	3.7±0.6	++	12.6±0.7
9	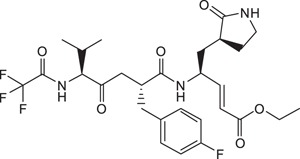	7.7±0.8	+++	32.7±6.7
10	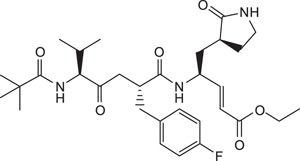	> 100	0	15.0±2.6
11	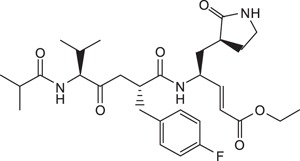	11.7±0.7	++	16.9±0.8

EV71 3 C protease biochemical inhibition potencies, 50% inhibitory concentration (IC_50_) was determined the protease inhibition assays. Cell-based antiviral activities was graded by the inhibitory activity detected at different concentrations (0: no activity detected; +, ++, +++ and ++++: activity detected at compound concentrations 5 μM, 1 μM, 100 nM and 10 nM respectively). Membrane permeability (P) was determined by parallel artificial membrane permeability assay (PAMPA).
