# Supplementary material for: Antiviral activities of peptide-based covalent inhibitors of the Enterovirus 71 3C protease
Source: Sci Rep. 2016 Sep 20;6:33663. doi: 10.1038/srep33663 (PMC5028882; doi:10.1038/srep33663)
Supplement: Supplementary Information [file srep33663-s1.pdf]

Antiviral activities of peptide-based covalent inhibitors of the Enterovirus 71 3C protease.

Yong Wah Tan[a], Melgious Jin Yan Ang[b], Qiu Ying Lau[b], Anders Poulsen[b], Fui Mee Ng[b], Siew Wen Then[b], Jianhe Peng[b], Jeffrey Hill[b], Wan Jin Hong[a], Cheng San Brian Chia[b]* and Justin Jang Hann Chu[a,c]*

[a]Institute of Molecular and Cell Biology, Agency for Science, Technology and Research (A*STAR), 61 Biopolis Drive, Proteos #06-05, Singapore 138673

[b]Experimental Therapeutics Centre, Agency for Science, Technology and Research (A*STAR), 31 Biopolis Way, Nanos #03-01, Singapore 138669

[c]Laboratory of Molecular RNA Virology and Antiviral Strategies, Department of Microbiology and Immunology, National University Health System, National University of Singapore, Singapore 117597

*Co-corresponding authors

Justin Chu

Tel: +65 65163278

E-mail: miccjh@nus.edu.sg

Brian Chia

Tel: +65 64070348

E-mail: cschia@etc.a-star.edu.sg

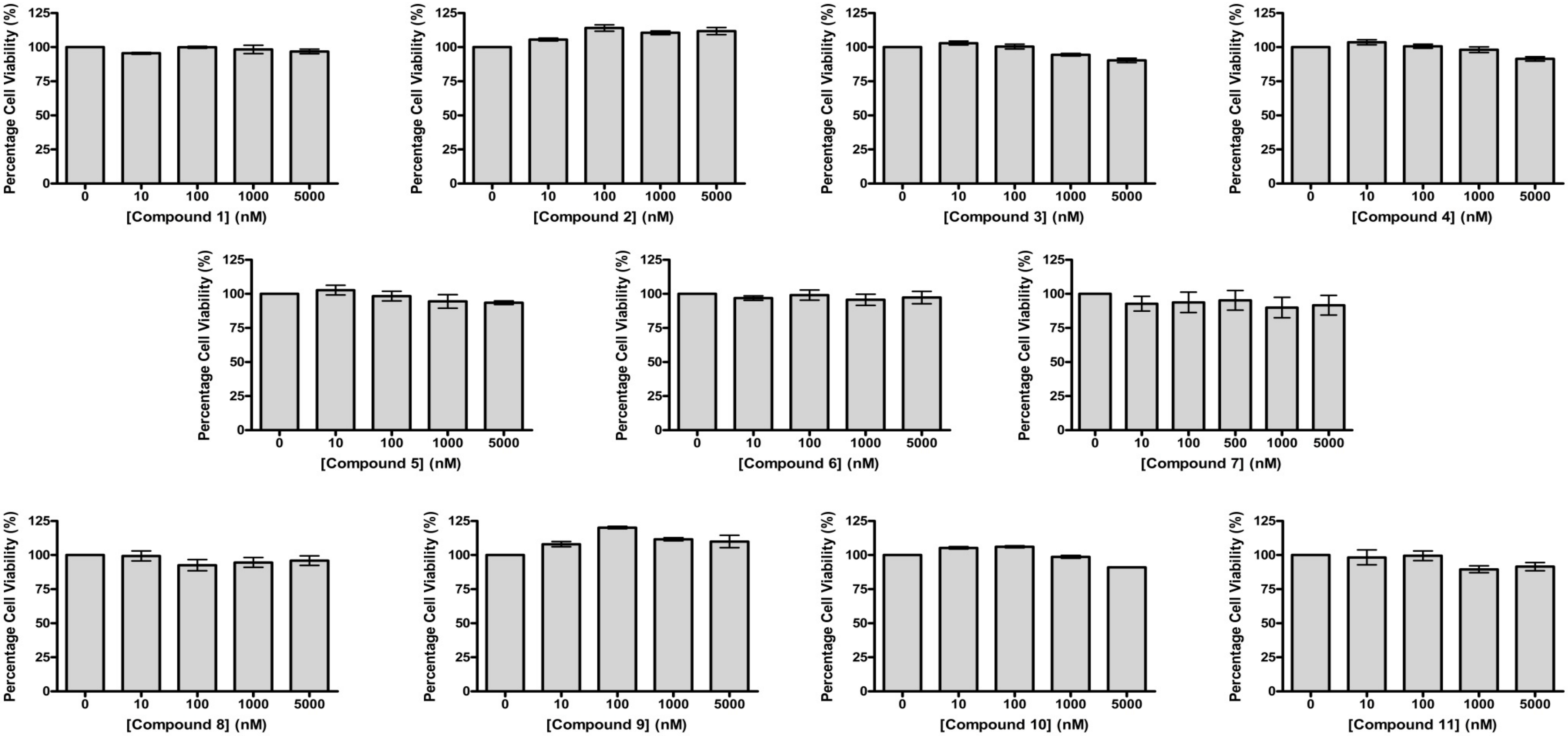

Supplementary Figure S1: Cellular viability of RD cells after treatment with compounds at different concentrations for 12 hours. Cellular viability was normalized to untreated (0 nM) samples and expressed as a percentage.
